# Efficacy and safety of deferasirox, an oral iron chelator, in heavily iron-overloaded patients with β-thalassaemia: the ESCALATOR study

**DOI:** 10.1111/j.1600-0609.2009.01228.x

**Published:** 2009-06

**Authors:** Ali Taher, Amal El-Beshlawy, Mohsen S Elalfy, Kusai Al Zir, Shahina Daar, Dany Habr, Ulrike Kriemler-Krahn, Abdel Hmissi, Abdullah Al Jefri

**Affiliations:** 1 American UniversityBeirut, Lebanon; 2 Cairo UniversityCairo, Egypt; 3 Ain Shams UniversityCairo, Egypt; 4 National Thalassemia CenterDamascus, Syrian Arab Republic; 5 Sultan Qaboos UniversityMuscat, Oman; 6 Novartis Pharmaceuticals CorporationNJ, USA; 7 Novartis Pharma AGBasel, Switzerland; 8 King Faisal Specialist Hospital and Research CenterRiyadh, Saudi Arabia

**Keywords:** iron chelation, deferasirox, β-thalassaemia, transfusional iron overload

## Abstract

**Objective:**

Many patients with transfusional iron overload are at risk for progressive organ dysfunction and early death and poor compliance with older chelation therapies is believed to be a major contributing factor. Phase II/III studies have shown that oral deferasirox 20–30 mg/kg/d reduces iron burden, depending on transfusional iron intake.

**Methods:**

The prospective, open-label, 1-yr ESCALATOR study in the Middle East was designed to evaluate once-daily deferasirox in patients ≥2 yr with β-thalassaemia major and iron overload who were previously chelated with deferoxamine and/or deferiprone. Most patients began treatment with deferasirox 20 mg/kg/d; doses were adjusted in response to markers of over- or under-chelation. The primary endpoint was treatment success, defined as a reduction in liver iron concentration (LIC) of ≥3 mg Fe/g dry weight (dw) if baseline LIC was ≥10 mg Fe/g dw, or final LIC of 1–7 mg Fe/g dw for patients with baseline LIC of 2 to <10 mg Fe/g dw.

**Results:**

Overall, 233/237 enrolled patients completed 1 yr’s treatment. Mean baseline LIC was 18.0 ± 9.1 mg Fe/g dw, while median serum ferritin was 3356 ng/mL. After 1 yr’s deferasirox treatment, the intent-to-treat population experienced a significant treatment success rate of 57.0% (*P* = 0.016) and a mean reduction in LIC of 3.4 mg Fe/g dw. Changes in serum ferritin appeared to parallel dose increases at around 24 wk. Most patients (78.1%) underwent dose increases above 20 mg/kg/d, primarily to 30 mg/kg/d. Drug-related adverse events were mostly mild to moderate and resolved without discontinuing treatment.

**Conclusions:**

The results of the ESCALATOR study in primarily heavily iron-overloaded patients confirm previous observations in patients with β-thalassaemia, highlighting the importance of timely deferasirox dose adjustments based on serum ferritin levels and transfusional iron intake to ensure patients achieve their therapeutic goal of maintenance or reduction in iron burden.

Regular blood transfusions from an early age are an essential therapy for patients with β-thalassaemia major in order to achieve optimal growth and adequate organ function, as well as to improve survival. As the human body cannot excrete excess iron, regularly transfused patients with β-thalassaemia will accumulate toxic, and eventually lethal levels of iron in the body. For more than 40 yr, deferoxamine (Desferal®; Novartis Pharma AG, Basel, Switzerland) has been the established chelation therapy for iron-overloaded patients, however the demanding infusion regimen often results in poor patient compliance.(1–3) Deferiprone (Ferriprox®; Apotex Inc., Toronto, ON, Canada) is a three-times daily, oral iron chelator that is available as a second-line therapy for β-thalassaemia patients for whom deferoxamine therapy is contraindicated or inadequate. Despite the availability of these chelating agents, some heavily transfused patients are unable to achieve successful iron chelation.

There remains a need for an effective and tolerable iron chelator with a less-demanding treatment schedule, which may help ensure long-term compliance in regularly transfused patients of all ages. Deferasirox is a once-daily, oral iron chelator approved for the treatment of transfusional iron overload in adult and paediatric patients. The efficacy and safety of deferasirox in patients with a variety of transfusion-dependent anaemias was established during five 1-yr Phase II/III clinical registration trials involving more than 1000 patients (4–8). The efficacy of deferasirox was monitored based on the monthly assessment of serum ferritin levels, which is the most widely used method for assessing body iron burden as it is convenient, relatively inexpensive and easily reproducible. As serum ferritin levels can be affected by factors such as inflammation (9) individual measurements should be interpreted with caution; however, serial measurements have been shown to be reliable for monitoring body iron burden. The deferasirox clinical trials demonstrated that the efficacy of treatment is dependent on dose, which should be tailored to the degree of iron burden and transfusional iron intake (10).

There is considerable evidence that iron burden should be closely monitored and an effective and tolerable long-term chelation therapy used in heavily transfused patients from an early age. The ESCALATOR trial was initiated to evaluate the efficacy and safety of deferasirox exclusively in regularly transfused patients with β-thalassaemia who had previously received deferoxamine and/or deferiprone therapy.

## Methods

### Inclusion criteria

Male or female patients (≥2 yr) with β-thalassaemia and transfusional iron overload were eligible for inclusion in the study if they had been treated with prior mono- or combination therapy with deferoxamine and/or deferiprone but had experienced unacceptable toxicity to deferoxamine, had poor response despite proper compliance with deferoxamine, had documented non-compliance of taking <50% of prescribed deferoxamine doses in the previous year or if deferoxamine treatment was contraindicated. Patients were also required to have a liver iron concentration (LIC) of ≥2 mg Fe/g dry weight (dw) and a serum ferritin level of ≥500 ng/mL. Paediatric patients have to be of adequate weight (9.4 kg in patients who received 20 mg/kg/d) to receive the smallest tablet of deferasirox.

Patients were excluded from the study if they had a mean alanine aminotransferase >300 U/L; serum creatinine above the upper limit of normal (ULN); significant proteinuria; uncontrolled hypertension; chronic hepatitis B or active hepatitis C receiving specific treatment; and a history of nephrotic syndrome, systemic diseases (cardiovascular, renal, hepatic) or any medical condition that may have affected absorption, distribution, metabolism or excretion of deferasirox. Patients were also excluded if they had been treated with any investigational drugs within the past 4 wk, or had a history of drug or alcohol abuse within the past 12 months or a history of non-compliance either with treatment or the protocol (e.g. patients who were considered potentially unreliable and/or not cooperative).

Institutional Review Board or Ethics Committee approval was obtained at each participating institution. All patients (or parents/guardians) provided written informed consent. The study was conducted in accordance with Good Clinical Practice guidelines and the Declaration of Helsinki.

### Study design

The ESCALATOR trial was a prospective, open-label, 1-yr, multicentre study conducted between June 2004 and November 2006 in seven thalassaemia treatment centres in the Middle East; data reported in this paper are based on patients from six sites. Patients were screened during a 56-d run-in period for eligibility criteria, and a baseline LIC was determined by liver biopsy. Patients previously receiving deferiprone discontinued treatment at least 28 d before receiving the first dose of study medication. Patients were permitted to receive deferoxamine until the day before they entered the study.

The initial protocol stated that patients should receive a deferasirox starting dose of 10 or 20 mg/kg/d, according to baseline LIC and serum ferritin. However, based on evidence from ongoing clinical trials indicating that 10 mg/kg/d was insufficient in heavily iron-overloaded patients, a subsequent protocol amendment introduced a fixed starting dose of 20 mg/kg/d. Routine dose adjustments were performed in response to markers of over- or under-chelation. Doses were increased based on serum ferritin trends (rises of ≥1000 ng/mL on two consecutive visits or >2500 ng/mL without decreasing trend) and reduced for elevated levels of serum creatinine, urinary protein : creatinine ratio and transaminases, and in response to adverse events such as skin rashes. Dose adjustments were performed in steps of 5 or 10 mg/kg/d, within a range of 0–30 mg/kg/d. If serum ferritin levels fell to ≤500 ng/mL at two consecutive study visits, deferasirox dose was withheld and resumed if serum ferritin rose to >1000 ng/mL. The transfusion goals of the study were to maintain a haemoglobin level of ≥9 g/dL, which was consistent from country to country.

### Efficacy assessments

The primary objective of the ESCALATOR study was to evaluate the effect of deferasirox treatment on LIC. LIC was determined by biopsy for all patients at baseline and following 1 yr of treatment. All measurements were performed in a central laboratory using atomic absorption spectrometry and were carried out according to standardized procedures. The primary efficacy endpoint was treatment success, defined as a reduction in LIC of ≥3 mg Fe/g dw after 1 yr of treatment with deferasirox if a patient’s baseline LIC was ≥10 mg Fe/g dw or a final LIC of 1–7 mg Fe/g dw if a patient’s baseline LIC was 2 to <7 or ≥7 to <10 mg Fe/g dw. Patients with a baseline LIC of ≥7 mg Fe/g dw had a therapeutic goal of LIC reduction, whilst in patients with a baseline LIC of 2 to <7 mg Fe/g dw, the therapeutic goal was maintenance.

Secondary efficacy endpoints included absolute and relative reduction in LIC from baseline to study end and total body iron excretion rate in subgroups defined by baseline LIC and age; transfusional iron intake and iron excretion were calculated according to the method previously described by Cohen *et al.*(10) Serum ferritin was assessed at baseline and at every 4 wk as a potential surrogate marker for LIC.

### Safety assessments

Safety was evaluated every 4 wk through the monitoring and recording of all adverse events and serious adverse events, and routine laboratory testing, including haematology, blood chemistry and urine renal function assessments. Liver specimens were graded by using the Ishak staging (measures fibrosis score) and grading (measures necroinflammatory score) system (11, 12). Left ventricular ejection fraction measurements by echocardiogram scan were also recorded. Physical and sexual development were evaluated in paediatric patients using height standard deviation score (13) (h-SDS) and Tanner stage assessments respectively.

### Statistical methods

For the intent-to-treat (ITT) analysis, all patients who had been successfully screened and chosen to start study treatment were included. All patients who received at least one dose of study medication were included in the safety population.

The efficacy analysis in this study was based on evaluation of ‘treatment success’ as described previously. Deferasirox therapy was considered to be effective if the treatment success rate was >50% in the ITT population. The null hypothesis was tested against the alternative using a *t*-test at a one-sided alpha level of 0.025 on the basis of the normal approximation of the binomial distribution. To examine the proportions of patients with a LIC of <15 mg Fe/g dw or serum ferritin <2500 ng/mL at baseline vs. 1 yr respectively, McNemar tests were performed whereby only patients with non-missing values at both visits were considered.

To demonstrate at an overall one-sided alpha level of 0.025 that the observed success rate is >50%, it was first calculated that 113 evaluable patients would be required to achieve a study power of 90%. However, in the ITT population, patients who dropped out of the trial were considered to be treatment failures so the attenuated success rate had to be accounted for by recruiting an estimated target of 250 patients, assuming an expected dropout rate of between 5% and 10%.

## Results

### Patient characteristics

Overall, 237 patients were enrolled from six centres: 162 paediatric patients (aged 2 to <16 yr) and 75 adults (aged ≥16 yr). A total of 233 (98.3%) patients completed 1 yr of treatment (Table 1). Of the four patients who discontinued, two patients were lost to follow-up and two patients died.

**Table 1:** Baseline patient characteristics

	Paediatric (*n*=162)	Adult (*n*=75)	All patients (*n*=237)
Mean age (range), yr	9.5 (2–15)	21.4 (16–42)	13.3 (2–42)
Female : male, *n*	80 : 82	37 : 38	117 : 120
Race (caucasian : oriental : other), *n*	59 : 82 : 21	11 : 43 : 21	70 : 125 : 42
1History of hepatitis B, *n* (%)	3 (1.9)	1 (1.3)	4 (1.7)
1History of hepatitis C, *n* (%)	44 (27.2)	29 (38.7)	73 (30.8)
Splenectomy, *n* (%)	46 (28.4)	53 (70.7)	99 (41.8)
Previous chelation therapy, *n* (%)			
Deferoxamine monotherapy	145 (89.5)	42 (56.0)	187 (78.9)
Deferiprone monotherapy	1 (0.6)	4 (5.3)	5 (2.1)
Deferoxamine + deferiprone	16 (9.9)	29 (38.7)	45 (19.0)
Mean no. of years receiving transfusions (SD)	8.6 (3.7)	18.9 (5.3)	11.8 (6.4)
Mean no. of transfusions in previous year prior to study entry (SD)	15.5 (4.4)	14.3 (3.7)	15.1 (4.2)
Total amount transfused, mL (SD)	5239 (2452)	7332 (2969)	5828 (2766)
Mean baseline LIC, mg Fe/g dw (SD)	17.0 (8.5)	20.1 (10.1)	18.0 (9.1)
Median baseline serum ferritin, ng/mL (range)	3326 (914–13 338)	3396 (956–25 008)	3356 (914–25 008)

LIC, liver iron concentration; dw, dry weight.

1 Investigator-reported patient history.

Baseline assessments of LIC and serum ferritin demonstrated that both the children (mean LIC: 17.0 ± 8.5 mg Fe/g dw; median serum ferritin: 3326 ng/mL) and adults (mean LIC: 20.1 ± 10.1 mg Fe/g dw; median serum ferritin: 3396 ng/mL respectively) enrolled in this study were primarily heavily iron overloaded despite previous chelation therapy.

### Dose adjustments

All patients started on deferasirox 20 mg/kg/d, except for three who started on 10 mg/kg/d, which was subsequently increased to 20 mg/kg/d based on protocol amendment. The median (range) average planned deferasirox dose was 23.1 (12–29) mg/kg/d, which patients received for a median (range) of 52.3 (6–61) wk. Deferasirox dose was adjusted in 192/237 (81.0%) patients. Most patients [185/237 (78.1%)] underwent dose increases above the 20 mg/kg/d starting dose. The most common dose increase was to 30 mg/kg/d [136 patients (57.4%)], while 47 (19.8%) patients increased to 25 mg/kg/d. The overall median (range) time to any dose increase was 26 (8–48) wk and the median (range) time to dose increase from 20 to 30 mg/kg/d was 24 (8–48) wk. Doses were reduced in nine patients (3.8%), all of which were due to serum creatinine increases.

A similar proportion of children and adults had dose adjustments: deferasirox dose was adjusted in 129 (79.6%) paediatric patients and 63 (84.0%) adults. The median (range) time to dose increase in the paediatric population was 30 (8–48) wk compared with 20 (8–44) wk in the adult population.

By the end of the study, most patients (75.9%; 55 adults, 125 children) were receiving ≥25 mg/kg/d deferasirox. Forty-seven (19.8%; 12 adults, 35 children) patients remained on 20 mg/kg/d deferasirox whilst six (2.5%, four adults, two children) were receiving 15 mg/kg/d.

### Effect of deferasirox on LIC

The ITT population experienced a statistically significant treatment success rate of 57.0% (95% confidence interval [CI] 50.7, 63.3; *P*=0.016). In the paediatric and adult populations, the treatment success rate was 57.4% (95% CI 49.8, 65.0) and 56.0% (95% CI 44.8, 67.2) respectively. Success rates in patients with baseline LIC of 2 to <7, ≥7 to <10 and ≥10 mg Fe/g dw were 72.7% (16/22), 50.0% (13/26) and 56.1% (106/189) respectively.

**Figure 1:**
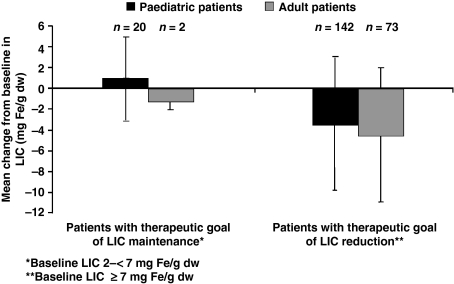
Mean change in liver iron concentration (LIC) after 1 yr’s treatment with deferasirox.

In patients whose therapeutic goal was LIC reduction, a statistically significant mean decrease in LIC of 3.8 ± 6.2 mg Fe/g dw (*P*<0.001) was achieved at study end (Fig. 1). In patients whose therapeutic goal was LIC maintenance, levels were maintained at approximately baseline level (mean overall increase of 0.9 ± 3.8 mg Fe/g dw; *P*=0.30, *post hoc* analysis) (Fig. 1). The mean absolute reduction in LIC from baseline in the ITT population was 3.4 mg Fe/g dw (*P*<0.001; adults: −4.4 ± 6.3 Fe/g dw; children: −2.9 ± 6.1 Fe/g dw).

Overall, 41.9% (*n* = 95) and 61.7% (*n* = 140) of patients had a LIC of <15 mg Fe/g dw at baseline and after 1 yr of treatment respectively; this was 44.0% (*n* = 70) and 60.1% (*n* = 97), and 36.8% (*n* = 25) and 63.2% (*n* = 43) in paediatric and adult patients respectively (*P*<0.0001 in all groups).

### Iron balance

Patients received a mean of 16.5 (±4.5) transfusions over the 1-yr study, receiving an average of 7.4 (±2.1) mL red blood cells/kg per transfusion. Paediatric patients received an average of 0.360 mL red blood cells/kg/d, resulting in an average iron intake of 0.388 mg/kg/d and an average iron excretion rate of 0.423 mg/kg/d. Thus, in the paediatric population, the iron excretion : iron intake ratio was 1.096, meaning only slightly more iron was excreted compared with intake from transfusions. The adult population had a positive excretion : intake ratio of 1.462. Adults received 0.260 mL red blood cells/kg/d, resulting in an average iron intake of 0.281 mg/kg/d and iron excretion rate of 0.392 mg/kg/d.

### Effect of deferasirox on serum ferritin

**Figure 2:**
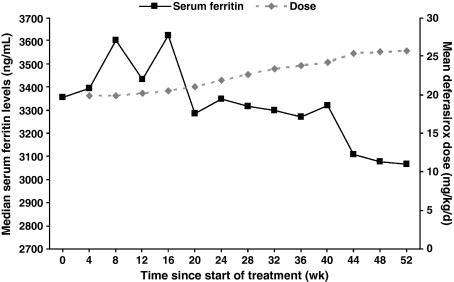
Median serum ferritin levels and mean deferasirox dose over the 1-yr study period.

In the ITT population, median serum ferritin decreased by 341 ng/mL (range: −9193 to 2835 ng/mL) at 52 wk (Fig. 2). Changes in serum ferritin appeared to parallel dose increases at around 24 wk in the adult population. A greater reduction in median serum ferritin was observed in the adult population than in the paediatric population during the course of this 1-yr study [adults: −846 ng/mL (range: −9193 to 2071 ng/mL); children: −166 ng/mL (range: −5071 to 2835 ng/mL)]. Overall, 35.8% (*n* = 83) and 40.5% (*n* = 94) of patients had serum ferritin levels <2500 ng/mL at baseline and after 1 yr of treatment respectively; this was 36.0% (*n* = 58) and 34.8% (*n* = 56), and 35.2% (*n* = 25) and 53.5% (*n* = 38) in paediatric and adult patients respectively (*P*=0.099 in the overall population and *P*<0.0002 in adults).

In patients whose therapeutic goal was reduction (baseline LIC ≥7 mg Fe/g dw, *n* = 215), a statistically significant median decrease in serum ferritin of 517 ng/mL (*P*<0.001) was achieved at study end. In patients whose therapeutic goal was maintenance (baseline LIC <7 mg Fe/g dw, *n* = 22), serum ferritin did not change significantly from baseline levels (median overall change of 289 ng/mL; *P*=0.302, *post hoc* analysis).

### Safety

Adverse events were reported by 180 (75.9%) patients overall, and the pattern of adverse events was comparable between the paediatric and adult populations. The most commonly reported adverse events, irrespective of the relationship to deferasirox treatment, included vomiting (11%), influenza (11%), nausea (8%), skin rash (8%), abdominal pain (6%) and diarrhoea (6%). Adverse events judged to be drug-related by the investigator were reported in 105 (44.3%) patients, most of which were mild to moderate in severity and resolved without the need for discontinuing treatment. The most common drug-related adverse events were vomiting, skin rash, nausea and increased alanine aminotransferase (Table 2). There were no discontinuations due to drug-related adverse events after the start of treatment.

**Table 2:** Most common (>3%) drug-related adverse events/laboratory abnormalities (*n*=237) in all patients

		Severity (%)		
Adverse event/laboratory abnormalities	Frequency (%)	Mild	Moderate	Severe
Vomiting	21 (8.9)	20 (8.4)	1 (0.4)	–
Skin rash	19 (8.0)	12 (5.1)	7 (3.0)	–
Nausea	17 (7.2)	15 (6.3)	2 (0.8)	–
Increased alanine aminotransferase^1^	13 (5.5)	8 (3.4)	4 (1.7)	1 (0.4)
Increased serum creatinine^1^	9 (3.8)	9 (3.8)	–	–

1 Assessed as an adverse event by the investigator.

Seventeen (7.2%) patients experienced a total of 36 serious adverse events, the most common of which were cholecystitis (1.6% of patients), splenomegaly (0.8%), cardiac failure (0.8%) and cholelithiasis (0.8%), pyrexia (0.4%), hypocalcaemia (0.4%) and renal failure (0.4%). Only one serious adverse event (obstructive jaundice of moderate severity) was considered by the investigator to be related to study treatment.

Two deaths from cardiac failure were reported; both were in adult patients with a history of poor compliance with all previous medications and neither was considered by the investigator to be related to study medication. One of these patients had a history of heart failure and died on day 269 of the study, while the other patient had a number of risk factors including atrial ectopics and type II diabetes and died on day 56 of the study. Serum ferritin levels in these patients at the time of death were 2193 and 9838 ng/mL respectively. Two patients were lost to follow-up.

Seventy-three patients [30.9%; 45 children (28.0%), 28 adults (37.3%)] had two consecutive serum creatinine levels >33% above baseline not exceeding the ULN; an additional six patients [2.5%; 4 children (2.5%), 2 adults (2.4%)] had two consecutive serum creatinine levels >33% above baseline and >ULN. Four patients (1.7%) experienced two consecutive increases in alanine aminotransferase >10 × ULN; of these three already had elevated alanine aminotransferase at baseline.

#### Ishak grading and staging

At baseline, the median (range) Ishak grade in ITT population was 2.0 (0–10) which decreased slightly to 1.0 (0–9) by the end of the study. In the paediatric and adult populations, median (range) Ishak grade decreased from 2.0 (0–10) and 1.0 (0–6) to 2.0 (0–9) and 1.0 (0–6) respectively. Mean change in each of three groups was −0.1, −0.2 and −0.2 respectively.

The median (range) Ishak stage in the ITT, paediatric and adult populations was 2.0 (0–6), 2.0 (0–6) and 3.0 (0–6) respectively. The mean change from baseline after 1 yr was 0.1 in the ITT population and the paediatric population and −0.1 in the adult population.

#### Cardiac function

There was a statistically significant increase in mean left ventricular ejection fraction in the ITT population over the course of the study, from 65.1 (±7.0) at baseline to 66.8 (±6.7) at week 52 (*P*=0.0002). Improvement in left ventricular ejection fraction appeared to be more pronounced in paediatric patients, with a mean (±SD) change from baseline of 2.5 (±8.3) in this population, compared with 0.4 (±6.8) in adults. There was no significant relationship between change in left ventricular ejection fraction vs. baseline serum ferritin (*P*=0.45) or change in left ventricular ejection fraction vs. change in serum ferritin (*P*=0.59).

#### Growth and development assessments in paediatric patients

**Figure 3:**
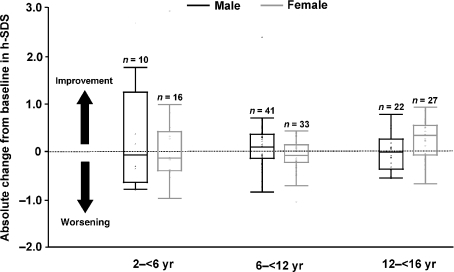
Absolute change from baseline in height standard deviation score in paediatric patients.

Body growth progressed normally in all paediatric patients (Fig. 3). Height comparisons with a CDC reference population (13) indicated that both boys and girls in this study were initially smaller than the reference group across all ages. Over the 1-yr study period, the observed growth as evaluated by changes from baseline in h-SDS showed growth improvements (>0.5 SDS) in 18.1% of patients, worsening in growth in 9.4% of patients, and no change from baseline in 72.5% of patients. In this population, girls aged 12 to <16 yr showed a notable improvement in growth, with 66.7% of this cohort exhibiting a net increase in h-SDS (25% percentile or first quartile = −0.06). Sexual development, as measured by Tanner stage assessments, also progressed normally.

## Discussion

The ESCALATOR trial conducted in the Middle East is the first study to evaluate the efficacy and safety of deferasirox exclusively in patients who were previously chelated with deferoxamine and/or deferiprone. It confirms the efficacy and safety of deferasirox in thalassaemia patients reported from a large randomized controlled study (6). Enrolled patients were characterized by a very high iron burden despite the previous chelation therapy. Mean LIC and median serum ferritin levels at baseline were above thresholds associated with significant negative outcomes (>15 mg Fe/g dw and >2500 ng/mL respectively), putting these patients at high risk for cardiac complications and death (14, 15).

According to prospectively defined criteria, the reduction or maintenance in iron burden achieved by patients receiving deferasirox in this study translated into a statistically significant treatment success rate of 57.0% (*P*=0.016), which is in-line with previous observations (6). In patients whose therapeutic goal was LIC reduction, a statistically significant mean decrease in LIC of 3.8 mg Fe/g dw (*P*<0.001) and serum ferritin of 517 ng/mL (*P*<0.001) was achieved at study end. Reducing LIC and serum ferritin in heavily iron-overloaded patients is essential for improving patient outcomes (16, 17). In patients whose therapeutic goal was LIC maintenance, LIC and serum ferritin levels were maintained around baseline. As most of these primarily heavily iron-overloaded patients did not receive a dose increase to ≥25 mg/kg/d until more than halfway through the 1-yr study, further decline in LIC and serum ferritin is expected with continued use of deferasirox treatment at a higher dose.

These data highlight the importance of timely deferasirox dose adjustments based on serum ferritin levels and transfusional iron intake to ensure patients achieve their therapeutic goal of maintenance or reduction in iron burden. Dose increases to 25 or 30 mg/kg/d were required in 78% of patients because they had not achieved their target reduction in iron burden. This is in line with previous studies demonstrating that a 20 mg/kg/d starting dose of deferasirox may not be sufficient to maintain or significantly reduce LIC and serum ferritin in heavily transfused, iron-overloaded patients, such as those in this study, although it is often adequate as a maintenance dose in less heavily transfused and iron-overloaded patients.(4–7) Furthermore, as dose adjustments occurred at a median of 24 wk the effect of deferasirox on LIC and serum ferritin at 52 wk may not reflect the full effect of receiving optimal doses of deferasirox for an extended period. An ongoing 2-yr extension phase of the ESCALATOR study is currently evaluating the efficacy of these optimal doses of deferasirox based on monthly serum ferritin measurements.

Reductions in serum ferritin levels and LIC were not as pronounced in the paediatric population as in the adult population. This may be because of a higher transfusional iron intake of children compared with adults (approximately 30% higher blood transfusions, 0.360 mL red blood cells/kg/d vs. 0.260 mL red blood cells/kg/d), and hence a higher iron intake (0.388 mg/kg/d vs. 0.281 mg/kg/d). There was also a longer median time to dose increases in the paediatric population (30 wk vs. 20 wk in adults). In addition, previous pharmacokinetic evaluation of deferasirox has shown that the exposure to deferasirox is lower in children than in adults.(4–6) Whether the differing efficacy results between the paediatric and adult population in this study are due to under-dosing, transfusional iron intake, the pharmacokinetic properties of deferasirox or the combination of these factors may be further elucidated during the extension phase. Further analyses evaluating the most appropriate deferasirox dosing strategy in paediatric patients would be of value, including chelation-naïve patients and those switching from other chelation therapies.

Deferasirox was generally well tolerated with a manageable safety profile over the course of the study. The adverse events observed in this 1-yr study were consistent with previous observations in patients with β-thalassaemia (4–6). There were no discontinuations due to drug-related adverse events, and no progressive increases in serum creatinine or liver transaminases. In particular, increased doses in the paediatric population did not result in any notable toxicity, and physical and sexual development progressed normally, showing significant improvement in adolescent girls. Growth retardation and hypogonadism remain significant clinical problems in adolescent patients with β-thalassaemia despite hypertransfusion and regular chelation therapy; these are encouraging results for a heavily transfused population (18, 19). The longer-term safety profile of deferasirox in these patients will be further assessed during the extension phase.

The risk of cardiac complications and early death are greatly increased in patients with β-thalassaemia who are heavily iron overloaded. Previous preliminary data (reported as an abstract) in patients with both decreased and normal baseline T2* values who were treated with deferasirox demonstrated an improvement in myocardial T2* after 1 yr of treatment; this improvement was maintained for 2 yr (20). In ESCALATOR, a statistically significant improvement in left ventricular ejection fraction was observed by week 52, suggesting a positive effect of deferasirox on cardiac function.

In conclusion, this study confirms previous observations in patients with β-thalassaemia (6). With appropriate dosing deferasirox controlled iron levels in this difficult-to-treat population of patients who were primarily heavily iron overloaded despite previous chelation with deferoxamine and/or deferiprone. The efficacy of deferasirox was dependent on transfusional intake and optimal dose, highlighting the importance of timely dose adjustment in order to achieve clinical goals. Deferasirox was generally well tolerated with no new adverse events when compared with the deferasirox registration trials.

## Participating centres*

Ali Taher: American University of Beirut-Chronic Care Center, Beirut, Lebanon. Amal El-Beshlawy: Cairo University, Cairo, Egypt. Mohsen S Elalfy: Ain Shams University, Cairo, Egypt. Kusai Al Zir: National Thalassemia Center, Damascus, Syrian Arab Republic. Shahina Daar: Sultan Qaboos University, Muscat, Oman. Abdullah Al Jefri: King Faisal Specialist Hospital and Research Center, Riyadh, Saudi Arabia. *King Abdulaziz University Hospital (*n* = 15) in Saudi Arabia was excluded from the final analysis since routine monitoring of study documents at the site could not confirm the accuracy of the data reported.
